# Developing a Survey Tool to Assess Implementation of Evidence-Based Chronic Disease Prevention in Public Health Settings Across Four Countries

**DOI:** 10.3389/fpubh.2019.00152

**Published:** 2019-06-11

**Authors:** Elizabeth L. Budd, Xiangji Ying, Katherine A. Stamatakis, Anna J. deRuyter, Zhaoxin Wang, Pauline Sung, Tahna Pettman, Rebecca Armstrong, Rodrigo Reis, Ross C. Brownson

**Affiliations:** ^1^Prevention Science Institute, College of Education, University of Oregon, Eugene, OR, United States; ^2^Prevention Research Center, Brown School, Washington University in St. Louis, St. Louis, MO, United States; ^3^College for Public Health and Social Justice, St. Louis University, St. Louis, MO, United States; ^4^Tongji University School of Medicine, Shanghai, China; ^5^Department of Applied Social Sciences, The Hong Kong Polytechnic University, Kowloon, China; ^6^Melbourne School of Population and Global Health, The University of Melbourne, Melbourne, VIC, Australia; ^7^School of Health and Biosciences, Pontifical Catholic University of Parana, Curitiba, Brazil

**Keywords:** chronic disease, reliability, evidence-based practice, implementation, international health

## Abstract

**Background:** Understanding the contextual factors that influence the dissemination and implementation of evidence-based chronic disease prevention (EBCDP) interventions in public health settings across countries could inform strategies to support the dissemination and implementation of EBCDP interventions globally and more effectively prevent chronic diseases. A survey tool to use across diverse countries is lacking. This study describes the development and reliability testing of a survey tool to assess the stage of dissemination, multi-level contextual factors, and individual and agency characteristics that influence the dissemination and implementation of EBCDP interventions in Australia, Brazil, China, and the United States.

**Methods:** Development of the 26-question survey included, a narrative literature review of extant measures in EBCDP; qualitative interviews with 50 chronic disease prevention practitioners in Australia, Brazil, China, and the United States; review by an expert panel of researchers in EBCDP; and test-retest reliability assessment.

**Results:** A convenience sample of practitioners working in chronic disease prevention in each country completed the survey twice (*N* = 165). Overall, this tool produced good to moderately reliable responses. Generally, reliability of responses was higher among practitioners from Australia and the United States than China and Brazil.

**Conclusions:** Reliability findings inform the adaptation and further development of this tool. Revisions to four questions are recommended before use in China and revisions to two questions before use in Brazil. This survey tool can contribute toward an improved understanding of the contextual factors that public health practitioners in Australia, Brazil, China, and the United States face in their daily chronic disease prevention work related to the dissemination and implementation of EBCDP interventions. This understanding is necessary for the creation of multi-level strategies and policies that promote evidence-based decision-making and effective prevention of chronic diseases on a more global scale.

## Introduction

Chronic diseases are a threat to global health, in developed and developing countries alike, accounting for 60% of deaths worldwide (1). The medical costs and loss of productivity related to chronic diseases are a great financial burden to individuals and economies (1). Evidence-based chronic disease prevention (EBCDP) interventions are effective tools for preventing chronic diseases (2). However, studies among U.S. and European public health practitioners indicate that only 56–64% of chronic disease prevention interventions currently in use are evidence-based (3, 4), while estimates of use of EBCDP interventions in lower and middle income countries are unknown. Studies in Australia and the United States have identified multi-level contextual factors that influence the dissemination and implementation (D&I) of EBCDP interventions. Examples of these contextual factors include individual- and agency-level capacity characterized by the training, structure, material and human resources at hand that hinder or facilitate the use of EBCDP interventions (2, 5–7). Additional work has addressed some of the contextual barriers by training practitioners on the evidence-based decision-making process, specifically clarifying the reasons for selecting EBCDP interventions and outlining how to find the interventions and resources to support effective implementation and quality improvement (3, 4, 7). These studies report increases in the D&I of EBCDP interventions among practitioners who attended the trainings. Research on Canadian public health departments has identified tailored messaging as an effective method for promoting the D&I of evidence-based interventions (8), and examined the pathways through which evidence is shared through organizational systems (9). These contextually specific findings inform next steps in addressing barriers and promoting evidence-based decision-making across the Canada. Little is known about these contextual factors that influence the D&I of EBCDP interventions in developing countries, nor the similarities and differences of contextual factors across countries. Several studies call for global strategies to improve the D&I of EBCDP interventions in order to more effectively reduce chronic diseases around the world (10–12). Reviews of measures used to assess the contextual factors that influence the D&I of EBCDP interventions highlight a lack of psychometric testing of the existing measures and room for improvement among those that have been tested (13–15). To assess cross-country contextual factors and inform globally-focused recommendations for facilitating the D&I of EBCDP interventions, a single survey tool that can be used across multiple, diverse countries is needed.

This study provides a detailed overview of the development and test-retest reliability of a survey tool to measure the stage of dissemination, multi-level contextual factors, and individual and agency characteristics that influence the D&I of EBCDP interventions in Australia, Brazil, China, and the United States. These countries were chosen for several reasons including, their leadership in distinct regions of the world (16–20), differences on contextual variables of interest (e.g., sociocultural, political/economic) (21), and high prevalence of chronic diseases (22). The World Health Organization reports from 2014 showed that the large majority of deaths in each of the four countries was due to chronic diseases (91% in Australia, 88% in the United States, 87% in China, and 74% in Brazil) (22). Further, based on the few studies of the D&I of EBCDP from Brazil and China (23, 24), compared with the many from Australia and the United States (25–29), Brazil, and China were selected as countries likely in earlier stages of dissemination of EBCDP than Australia and the United States.

## Materials and Methods

### Survey Tool Development

**Figure 1:**
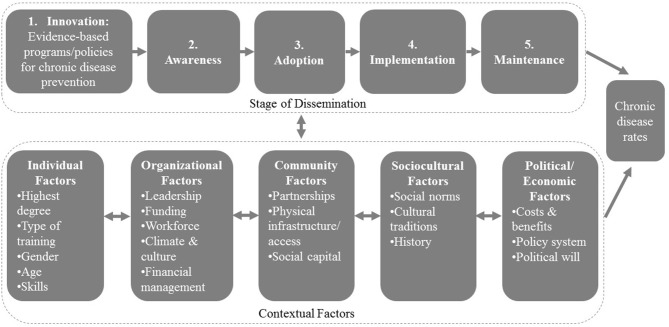
Factors affecting the stages of dissemination of evidence-based programs and policies for chronic disease prevention.

Development of the 26-question survey occurred in several stages. First, a guiding framework was developed based on previous work (30, 31) of the research team (see Figure 1). This framework informed subsequent stages of survey tool development, ensuring that qualitative interview questions and initial survey drafts were literature-based and comprehensive from the outset.

Second, a narrative literature review of extant measures in EBCDP was carried out in order to identify relevant questions and gaps in the D&I of EBCDP literature (2, 6, 31–35). Third, between February and July 2015 semi-structured interviews of public health practitioners in Australia (*n* = 13), Brazil (*n* = 9), China (*n* = 16), and the United States (*n* = 12) were conducted by trained researchers. Practitioners were identified through purposive sampling based on their employment at agencies responsible for the prevention of chronic disease in each country, including community health services, regional health departments, and non-government organizations (Australia); the ministry of health and local health departments (Brazil); hospitals, community health centers, and the Centers for Disease Control and Prevention (China); and local health departments (United States). The interviews were performed in English, Chinese, or Portuguese, audio recorded, transcribed, translated to English by two bi-lingual research team members (*n* = 25) when appropriate, and analyzed using deductive, hierarchical coding in NVivo version 10.

Forth, drafts of the survey underwent expert review by 13 chronic disease prevention researchers and were translated forward and backward to Chinese and Portuguese from English. Survey questions were organized into one of the five stages of dissemination or as multi-level contextual factors seen in Figure 1. Individual and agency characteristics were also included. Seven response items were deemed non-applicable or inappropriate for China contexts, but were included in the survey for the other three countries. These response items and the resulting tool can be found in Table 1.

**Table 1:** Factors influencing the dissemination and implementation of evidence-based chronic disease prevention across four countries: a survey tool.

**Questions**	**Response options**
**Awareness**	
Evidence-based public health is defined as: “the process of integrating science-based interventions with community preferences to improve the health of populations” (36). 1. With this definition in mind, how knowledgeable are you with evidence-based processes? (*select one*)	Not at all knowledgeable Slightly knowledgeable Somewhat knowledgeable Moderately knowledgeable Extremely knowledgeable
**Adoption**	
Definition: Evidence-based interventions are those that several studies have found to be effective at preventing chronic disease. Repositories are collections of evidence-based interventions (e.g., Guide to Community Preventive Services) (US), Health-Evidence.org (Australia), Cochrane Collaboration (US, Australia). 2. I have used repositories to find evidence-based interventions: (*select one*)	In none of my programmatic areas In a few of my programmatic areas In many of my programmatic areas In all of my programmatic areas
3. Staff at my agency use repositories of evidence-based interventions: (*select one*)	In none of my programmatic areas In a few of my programmatic areas In many of my programmatic areas In all of my programmatic areas
4. When you make decisions about such things as program planning and implementation, policy development, or funding, which of the following are important to you? (*select the top three*)	Support from leadership at my agency Support from elected officials Support from community partnerships Recommendations from the funding agency Colleagues are using the intervention Available resources (program dollars and staff) How easy the intervention or policy is to implement Evidence regarding the effectiveness of the intervention Health planning tools (e.g., MAPP or Health People 2010) Relevance of the intervention to the population of interest Seriousness of the health problem Other, please specify ______ Not applicable
5. What avenues do you use to learn about the current study findings on evidence-based chronic disease prevention interventions? (*select all that apply*)	Academic journals Conferences Email alerts Evidence-based repositories Facebook Funders^a^ Government agency staff Government reports Internet search engines Listservs/Newsletters/Online forums Media campaigns/Media interviews Networks Partnerships (e.g., with universities, health departments, professional associations) Policy briefs^a^ Press releases Stakeholders^a^ Technical assistance/Data liaison Trainings/Workshops/Meetings within my agency Webinars Other, please specify ______ None
6. For which avenues would you like additional access? (*select all that apply*)	*Same responses as #13*
**Implementation**	
7. Approximately what percentage of programs supported by your agency would you say are evidence-based?	*Fill in the blank 0–100%*
8. As you think about the future, what is one thing you would change to help you implement evidence-based chronic disease prevention interventions?	*Fill in the blank*
**Maintenance**	
Quality improvement (QI) refers to ongoing formal assessments of the effectiveness and quality of public health chronic disease prevention efforts. (37).Some examples of quality improvement processes include*: R*esults-based accountability (RBA), Community Health Improvement Plan (CHIP), Plan-Do-Study-Act (PDSA), and Plan-Do-Check-Act. 9. Staff at my agency use quality improvement processes: (*select one*)	In none of my programmatic areas In a few of my programmatic areas In many of my programmatic areas In all of my programmatic areas
10. In your opinion, how often do programs end that should have continued? (i.e., end without warrant) (*select one*)	Never Sometimes Often
11. When you think about public health programs that have ended, what are the most common reasons for programs ending? (*Select the top three*)	Program was never evaluated Program was evaluated but did not demonstrate impact Opposition/lack of support from leaders in my agency Opposition/lack of support from the general public Opposition/lack of support from policy makers Funding diverted to a higher priority program Grant funding ended Change in political leadership Insurance funding/coverage ended Program was adopted or continued by other organizations A program champion departed Program was not evidence-based Program was expensive Program was challenging to maintain Other, please specify ______ I do not know Not applicable
12. In your opinion, how often do programs continue that should have ended? (i.e., continue without warrant) (*select one*)	Never Sometimes Often
13. When you think about public health programs that continued that should have ended, what are the most common reasons for their continuation? (i.e., continue without warrant) (*Select the top three*)	Program was never evaluated Sustained support from leaders in your agency Sustained support from the general public Sustained support from policymakers Prohibitive costs of starting something new Absence of alternative options Sustained funding Presence of a program champion Program was considered evidence-based Program was low-cost Program was easy to maintain Other, please specify ______ I do not know Not applicable
**Contextual factors**	
14. Which of the following are personal barriers that make it harder for you to select and implement evidence-based chronic disease prevention interventions? *(Select all that apply*)	Not being an expert on relevant issues Lack of confidence in finding data and statistics Lack of skills to develop evidence-based interventions Lack of confidence in carrying out evidence-based interventions Lack of decision-making authority Low value of evidence-based approaches Workload is too heavy/not enough time Overwhelmed by task Other, please specify ______ None
15. Which of the following are agency-level barriers that make it harder for you to select and implement evidence-based chronic disease prevention interventions? (*Select all that apply*)	Poor understanding of evidence-based approaches Culture/climate is not supportive of change/new ideas No existing policies to support evidence-based approaches Agency does not provide training in evidence-based approaches Staff/leaders lack formal training in evidence-based approaches Lack of access to resources (e.g., computer, Internet) Not enough funding Low priority placed on chronic disease prevention No systems to ensure interventions are evidence-based Not enough staff Beliefs that evidence-based interventions are too difficult to implement/sustain Other, please specify ______ None
16. Which of the following are community-level barriers that make it harder for you to select and implement evidence-based chronic disease prevention interventions? (*Select all that apply*)	Lack of access to repositories/databases of scientific studies Lack of partnership between agency and community Community members' needs compete with evidence-based recommendations Catering to preferences of funders^a^ Low priority placed on chronic disease prevention Other, please specify ______ None
17. Which of the following are sociocultural barriers that make it harder for you to select and implement evidence-based chronic disease prevention interventions? (*Select all that apply*)	Distrust of scientific data in the populations served Community cultural practices conflict with evidence-based recommendations Not enough relevant evidence for populations served Serving a rural setting where data are lacking^a^ Serving a highly disadvantaged population Serving a population that speaks a language different from the majority^a^ Evidence is presented in a language I do not understand Other, please specify ______ None
18. Which of the following are political/economic barriers that make it harder for you to select and implement evidence-based chronic disease prevention interventions? (*Select all that apply*)	Political leaders not providing enough support Funding changes that occur with changes in political leadership Political climate conflicts with evidence-based chronic disease prevention recommendations Health care system does not support evidence-based chronic disease prevention Other, please specify ______ None
19. For which of the following skills would you like additional technical support or training? (*Check all that apply*)	Prioritizing program and policy options Quantifying the public health issue using descriptive epidemiology (e.g., concepts of person, place, time) Using quantitative evaluation approaches (e.g., surveillance or surveys) Using qualitative evaluation approaches (e.g., focus groups, key informant interviews) Developing an action plan for achieving goals Defining the health issue according to the community's needs and assets Adapting interventions for different communities and settings Using economic data in the decision making process Communicating research to policy makers Other, please specify ________ None
**Individual and agency characteristics**	
20. What is your gender? (*select one*)	Male Female Other Prefer not to answer
21. What is your age? (*select one*)	21–29 30–39 40–49 50–59 60 and over Prefer not to answer
22. What degree/credentials do you hold? (*Check all that apply*)	BS/BA CHES Certified Health Educator (in Diabetes, Asthma, etc.) RN or RD MS or MSc MPH or MSPH MA Other Master's degree NP MO or DO PhD, DrPH, ScD Other, please specify ______
23. Though you may work in several capacities, how do you best describe your primary position? (*select one*)	Academic Researcher Academic Educator Community Health Nurse Department Head Division or Bureau Head/ Division Deputy Director Epidemiologist Health Educator Nutritionist/Dietician Physician Program Manager/Administrator/Coordinator Program Planner/ Evaluator Public Health Specialist Social Worker Statistician Other, please specify ______
24. The agency in which I work has the following number of employees. (*select one*)	0–50 51–100 101–200 201–400 401–800 >800 I do not know
25. The size of the population my agency serves is has the following number of people. (*select one*)	0–24,999 25,000–49,999 50,000–74,999 75,000–99,999 100,000–149,999 150,000–199,999 200,000–299,999 300,000–399,999 400,000+ I do not know
26. Is there anything else you would like to share on the topic of evidence-based chronic disease prevention? Please specify	*Fill in the blank*

a *This item was not applicable and not included in the survey for respondents in China*.

Fifth, research team members in each country recruited public health practitioners working in chronic disease prevention, primarily on the local and regional levels, in each of the four countries to complete the survey. Samples of practitioners from various regions of each country were identified through national databases and networks of chronic disease prevention practitioners between November 2015 and April 2016. Public health systems across countries varied so much that there was no equivalent sampling method that worked for all four countries. In the United States, a stratified (by region) random sample of chronic disease prevention practitioners from a national database received up to three emails and two follow-up telephone calls requesting participation in the electronic survey (58% response rate). In Australia, up to two emails requesting participation in the electronic survey were sent to all chronic disease practitioners in a national registry (18% response rate). In Brazil, the same protocol as was followed in the United States was used, but with an additional follow-up telephone call (46% response rate). In China, a convenience sample of practitioners working within a network of community hospitals received one email and one follow-up telephone call requesting participation in the electronic survey (87% response rate). All surveys were delivered by an email embedded link and completed electronically. Upon completion of the survey, all respondents were asked to re-take the survey two to three weeks later for test-retest reliability testing purposes. This process was repeated until each respondent to the survey had been contacted twice, requesting them to retake the survey. Calculating Cohen's kappa and Intraclass correlation coefficients (ICC) ranging from 0.50 to 0.70 require a sample size of 25–50 test-retest pairs, respectively (38), thus 25 pairs were the minimum, but 50 pairs were the goal. During data collection, political events in Brazil affected the work lives of many Brazilian chronic disease practitioners and made recruitment of Brazilian practitioners extraordinarily difficult (39, 40). The data collection period was extended for research team investigators in Brazil in order to reach the minimum sample size.

This study was carried out in accordance with the committee responsible for human experimentation (institutional and national) and with the World Medical Association's Declaration of Helsinki with informed consent from all subjects. After reading the electronic informed consent document, subjects indicated their consent by selecting a radial button at the bottom of the informed consent document that read, “I consent to participate in this research study.” Additional written documentation of consent was waived and the protocol was approved by The University of Melbourne Human Ethics Committee, Pontifica Universidade Catolica do Parana Research Ethics Committee, The Hong Kong Polytechnic University Human Ethics Committee of the Faculty of Health and Social Science, and Washington University in St. Louis Institutional Review Board.

### Analyses

Test-retest reliability was examined on the survey questions, excluding open-ended questions and individual and agency characteristics. Intraclass correlation coefficients (ICC) were calculated for questions with ordinal response options (questions 1 through 3, 9, 10, and 12; see Table 1). “I don't know” and “not applicable” response options were not included in the ICC calculations. Each response item for questions 4, 5, 11, and 13 through 19 was dichotomized to reflect whether a respondent selected the response option or not. Cohen's kappa was run for each of these response options individually. The mean of all of the Cohen's kappas for each question's set of response options was calculated. Cut-points for ICC and mean kappa (excellent: ≥0.801; good: 0.601–0.80; moderate: 0.401–0.60; poor: ≤0.40) were selected based on recommendations (41, 42), and to aid in the interpretation of the results. Percentage agreement was also calculated for all of the aforementioned questions, excluding question 7, which asked respondents to provide a percentage. Questions for which mean kappa was calculated, mean percentage agreement was also calculated. Cut-points for percentage agreement included: excellent: 89.5–100%; good: 74.5–89.4%; moderate: 60–74.4%; and poor: <60%. All analyses were conducted in Stata version 14.

## Results

There were 400 survey respondents total and 165 of them took the survey twice for test-retest reliability purposes (*N* = 39 from Australia; *N* = 27 from Brazil; *N* = 45 from China; *N* = 54 from the United States). The test-retest respondents were all public health practitioners (e.g., nutritionist/dietician, coordinator, community health nurse) working in chronic disease prevention. Public Health Specialist was added as a primary employment position option *post hoc*, in order to capture a common “other” response provided by practitioners from Brazil. Respondents were primarily female (79%) between 30 and 49 years old (53%). The mean survey completion time varied by country, with Brazil having the longest (33.2 min ± 27.8), followed by the United States (17.72 min ± 13.4), Australia (16.6 min ± 10.0), and China (13.8 min ± 10.5). The mean number of days between test and retest was greatest in Brazil (46.4 ± 28.5), followed by Australia (39.0 ± 2.8), China (23.7 ± 7.6) and the United States (21.0 ± 9.1). Table 2 shows frequency counts for each response option by country, the first time respondents completed the survey. Item responses vary in prevalence from zero endorsements to endorsement from a large majority of a county's sample.

**Table 2:** Frequency of response option endorsement by country (*N* = 165).

**Question and response options**	**Australia** **(Total *N* = 39)**	**Brazil** **(Total *N* = 27)**	**China** **(Total *N* = 45)**	**United States** **(Total *N* = 54)**
	***N* (%)**	***N* (%)**	***N* (%)**	***N* (%)**
1. How knowledgeable are you with evidence-based processes?				
Not at all knowledgeable	0 (0.0)	0 (0.0)	7 (15.6)	0 (0.0)
Slightly knowledgeable	0 (0.0)	1 (3.7)	15 (33.3)	1 (1.9)
Somewhat knowledgeable	9 (23.1)	8 (29.6)	16 (35.6)	6 (11.1)
Moderately knowledgeable	22 (56.4)	13 (48.1)	8 (17.8)	31 (57.4)
Extremely knowledgeable	8 (20.5)	5( 18.5)	1 (2.2)	16 (29.6)
2. I have used repositories to find evidence-based interventions:				
In none of my programmatic areas	2 (5.1)	0 (0.0)	12 (26.7)	3 (5.6)
In a few of my programmatic areas	11 (28.2)	7 (25.9)	25 (55.6)	18 (33.3)
In many of my programmatic areas	16 (41.0)	7 (25.9)	6 (13.3)	27 (50.0)
In all of my programmatic areas	8 (20.5)	13 (48.1)	1 (2.2)	5 (9.3)
I don't know	0 (0.0)	0 (0.0)	0 (0.0)	0 (0.0)
Not applicable	2 (5.13)	0 (0.0)	3 (6.7)	1 (1.9)
3. Staff at my agency use repositories of evidence-based interventions:				
In none of their programmatic areas	0 (0.0)	0 (0.0)	11 (24.4)	2 (3.7)
In a few of their programmatic areas	9 (23.1)	5 (18.5)	19 (42.2)	18 (33.3)
In many of their programmatic areas	19 (48.7)	11 (40.7)	8 (17.8)	24 (44.4)
In all of their programmatic areas	4 (10.3)	9 (33.3)	0 (0.0)	1 (1.9)
I don't know	3 (7.7)	1 (3.7)	9 (20.0)	7 (13.0)
Not applicable	3 (7.7)	1 (3.7)	0 (0.0)	2 (3.7)
4. When you make decisions about such things as program planning and implementation, policy development, or funding, which of the following are important to you?				
Support from leadership at my agency	9 (23.1)	22 (81.5)	31 (68.9)	24 (44.4)
Support from elected officials	5 (12.8)	13 (48.1)	17 (37.8)	4 (7.4)
Support from community partnerships	13 (33.3)	20 (74.1)	16 (35.6)	21 (38.9)
Recommendations from the funding agency/ Recommendations from the Research Management Department (China)	1 (2.6)	17 (63.0)	10 (22.2)	16 (29.6)
Colleagues are using the intervention	1 (2.6)	19 (70.4)	6 (13.3)	1 (1.9)
Available resources (program dollars and staff)	15 (38.5)	26 (96.3)	25 (55.6)	36 (66.7)
How easy the intervention or policy is to implement	2 (5.1)	12 (44.4)	23 (51.1)	1 (1.9)
Evidence regarding the effectiveness of the intervention	30 (76.9)	23 (85.2)	13 (28.9)	32 (59.3)
Health planning tools (e.g. MAPP or Health People 2010)/Government Health plans (China)	5 (12.8)	21 (77.8)	9 (20.0)	1 (1.9)
Relevance of the intervention to the population of interest	26 (66.7)	24 (88.9)	18 (40.0)	20 (37.0)
Seriousness of the health problem	9 (23.1)	20 (74.1)	9 (20.0)	5 (9.3)
Other	1 (2.6)	0 (0.0)	0 (0.0)	1 (1.9)
Not applicable	0 (0.0)	0 (0.0)	1 (2.2)	0 (0.0)
5. What avenues do you use to learn about the current study findings on evidence-based chronic disease prevention interventions?				
Academic journals	36 (92.3)	19 (70.4)	23 (51.1)	24 (44.4)
Conferences	35 (89.7)	14 (51.9)	14 (31.1)	36 (66.7)
Email alerts	28 (71.8)	5 (18.5)	5 (11.1)	32 (59.3)
Evidence-based repositories	17 (43.6)	19 (70.4)	8 (17.8)	31 (57.4)
Facebook/Weibo, Wechat (China)	4 (10.3)	1 (3.7)	10 (22.2)	4 (7.4)
Funders^a^	4 (10.3)	0 (0.0)	–	23 (42.6)
Government agency staff	12 (30.8)	17 (63.0)	2 (4.4)	25 (46.3)
Government reports	23 (59.0)	23 (85.2)	3 (6.7)	23 (42.6)
Internet search engines	21 (53.8)	16 (59.3)	14 (31.1)	32 (59.3)
Listservs/Newsletters/Online forums	15 (38.5)	2 (7.4)	3 (6.7)	25 (46.3)
Media campaigns/Media interviews	4 (10.3)	3 (11.1)	6 (13.3)	7 (13.0)
Networks	23 (59.0)	10 (37.0)	5 (11.1)	18 (33.3)
Partnerships (e.g., with universities, health departments, professional associations)	26 (66.7)	14 (51.9)	7 (15.6)	35 (64.8)
Policy briefs^a^	11 (28.2)	12 (44.4)	–	17 (31.5)
Press releases	8 (20.5)	3 (11.1)	6 (13.3)	9 (16.7)
Stakeholders^a^	11 (28.2)	27 (100.0)	–	14 (25.9)
Technical assistance/Data liaison	1 (2.6)	13 (48.1)	2 (4.4)	21 (38.9)
Trainings/Workshops/Meetings within my agency	17 (43.6)	11 (40.7)	18 (40.0)	7 (13.0)
Webinars	16 (41.0)	2 (7.4)	1 (2.2)	36 (66.7)
Other	0 (0.0)	2 (7.4)	0 (0.0)	1 (1.9)
None	1 (2.6)	1 (3.7)	5 (11.1)	0 (0.0)
6. For which avenues would you like additional access?				
Academic journals	12 (30.8)	16 (59.3)	17 (37.8)	12 (22.2)
Conferences	8 (20.5)	17 (63.0)	10 (22.2)	16 (29.6)
Email alerts	3 (7.7)	5 (18.5)	10 (22.2)	4 (7.4)
Evidence-based repositories	13 (33.3)	7 (25.9)	19 (42.2)	18 (33.3)
Facebook/Weibo, Wechat (China)	2 (5.1)	2 (7.4)	12 (26.7)	3 (5.6)
Funders^a^	4 (10.3)	3 (11.1)	–	10 (18.5)
Government agency staff	6 (15.4)	2 (7.4)	2 (4.4)	6 (11.1)
Government reports	8 (20.5)	5 (18.5)	8 (17.8)	3 (5.6)
Internet search engines	1 (2.6)	2 (7.4)	11 (24.4)	3 (5.6)
Listservs/Newsletters/Online forums	4 (10.3)	3 (11.1)	14 (31.1)	8 (14.8)
Media campaigns/Media interviews	2 (5.1)	1 (3.7)	4 (8.9)	4 (7.4)
Networks	9 (23.1)	6 (22.2)	2 (4.4)	8 (14.8)
Partnerships (e.g., with universities, health departments, professional associations)	13 (33.3)	11 (40.7)	13 (28.9)	15 (27.8)
Policy briefs	5 (12.8)	2 (7.4)	0 (0.0)	4 (7.4)
Press releases	2 (5.1)	2 (7.4)	7 (15.6)	2 (3.7)
Stakeholders^a^	4 (10.3)	2 (7.4)	–	3 (5.6)
Technical assistance/Data liaison	6 (15.4)	1 (3.7)	9 (20.0)	12 (22.2)
Trainings/Workshops/Meetings within my agency	8 (20.5)	10 (37.0)	18 (40.0)	12 (22.2)
Webinars	10 (25.6)	1 (3.7)	3 (6.7)	9 (16.7)
Other	3 (7.7)	0 (0.0)	0 (0.0)	0 (0.0)
None	1 (2.6)	0 (0.0)	3 (6.7)	0 (0.0)
Questions 7 and 8 N/A				
9. Staff at my agency use quality improvement processes:				
In none of their programmatic areas	0 (0.0)	1 (3.7)	9 (20.0)	0 (0.0)
In a few of their programmatic areas	10 (25.6)	8 (29.6)	16 (35.6)	20 (37.0)
In many of their programmatic areas	16 (41.0)	12 (44.4)	15 (33.3)	28 (51.9)
In all of their programmatic areas	8 (20.5)	5 (18.5)	1 (2.2)	4 (7.4)
I don't know	3 (7.7)	1 (3.7)	5 (11.1)	1 (1.9)
Not applicable	2 (5.1)	0 (0.0)	0 (0.0)	1 (1.9)
10. In your opinion, how often do programs end that should have continued?				
Never	2 (5.1)	21 (77.8)	21 (46.7)	0 (0.0)
Sometimes	15 (38.5)	2 (7.4)	2 (4.4)	28 (51.9)
Often	20 (51.3)	3 (11.1)	19 (42.2)	24 (44.4)
11. When you think about public health programs that have ended, what are the most common reasons for programs ending?				
Program was never evaluated	9 (23.1)	9 (33.3)	3 (6.7)	9 (16.7)
Program was evaluated but did not demonstrate impact	12 (30.8)	8 (29.6)	16 (35.6)	7 (13.0)
Opposition/lack of support from leaders in my agency	7 (17.9)	10 (37.0)	8 (17.8)	6 (11.1)
Opposition/lack of support from the general public	1 (2.6)	6 (22.2)	20 (44.4)	6 (11.1)
Opposition/lack of support from policy makers	10 (25.6)	8 (29.6)	10 (22.2)	10 (18.5)
Funding diverted to a higher priority program	11 (28.2)	13 (48.1)	12 (26.7)	20 (37.0)
Grant funding ended	25 (64.1)	14 (51.9)	12 (26.7)	46 (85.2)
Change in political leadership	17 (43.6)	15 (55.6)	3 (6.7)	4 (7.4)
Insurance funding/coverage ended	1 (2.6)	4 (14.8)	0 (0.0)	6 (11.1)
Program was adopted or continued by other organizations	0 (0.0)	1 (3.7)	0 (0.0)	8 (14.8)
A program champion departed	9 (23.1)	9 (33.3)	3 (6.7)	1 (1.9)
Program was not evidence-based	1 (2.6)	7 (25.9)	5 (11.1)	3 (5.6)
Program was expensive	1 (2.6)	4 (14.8)	8 (17.8)	11 (20.4)
Program was challenging to maintain	2 (5.1)	4 (14.8)	24 (53.3)	2 (3.7)
Other, please specify ______	2 (5.1)	0 (0.0)	1 (2.2)	2 (3.7)
I do not know	0 (0.0)	0 (0.0)	4 (8.9)	1 (1.9)
Not applicable	1 (2.6)	1 (3.7)	0 (0.0)	5 (9.3)
12. In your opinion, how often do programs continue that should have ended?				
Never	1 (2.6)	4 (14.8)	19 (42.2)	0 (0.0)
Sometimes	20 (51.3)	21 (77.8)	2 (4.4)	35 (64.8)
Often	10 (25.6)	2 (7.4)	22 (48.9)	13 (24.1)
13. When you think about public health programs that continued that should have ended, what are the most common reasons for their continuation?				
Program was never evaluated	12 (30.8)	7 (25.9)	6 (13.3)	9 (16.7)
Sustained support from leaders in your agency	13 (33.3)	6 (22.2)	14 (31.1)	16 (29.6)
Sustained support from the general public	6 (15.4)	5 (18.5)	17 (37.8)	8 (14.8)
Sustained support from policymakers	11 (28.2)	12 (44.4)	15 (33.3)	21 (38.9)
Prohibitive costs of starting something new	9 (23.1)	4 (14.8)	5 (11.1)	5 (9.3)
Absence of alternative options	9 (23.1)	7 (25.9)	13 (28.9)	9 (16.7)
Sustained funding	7 (17.9)	12 (44.4)	14 (31.1)	26 (48.1)
Presence of a program champion	12 (30.8)	7 (25.9)	5 (11.1)	13 (24.1)
Program was considered evidence-based	4 (10.3)	2 (7.4)	9 (20.0)	5 (9.3)
Program was low-cost	9 (23.1)	6 (22.2)	5 (11.1)	11 (20.4)
Program was easy to maintain	10 (25.6)	6 (22.2)	12 (26.7)	15 (27.8)
Other, please specify ______	4 (10.3)	1 (3.7)	0 (0.0)	4 (7.4)
I do not know	0 (0.0)	3 (11.1)	5 (11.1)	1 (1.9)
Not applicable	2 (5.1)	0 (0.0)	3 (6.7)	0 (0.0)
14. Which of the following are personal barriers that make it harder for you to select and implement evidence-based chronic disease prevention interventions?				
Not being an expert on relevant issues	10 (25.6)	5 (18.5)	29 (64.4)	12 (22.2)
Lack of confidence in finding data and statistics	7 (17.9)	1 (3.7)	10 (22.2)	6 (11.1)
Lack of skills to develop evidence-based interventions	6 (15.4)	6 (22.2)	18 (40.0)	8 (14.8)
Lack of confidence in carrying out evidence-based interventions	3 (7.7)	1 (3.7)	7 (15.6)	3 (5.6)
Lack of decision-making authority	23 (59.0)	8 (29.6)	20 (44.4)	15 (27.8)
Low value of evidence-based approaches	5 (12.8)	13 (48.1)	0 (0.0)	1 (1.9)
Workload is too heavy/not enough time	19 (48.7)	5 (18.5)	19 (42.2)	33 (61.1)
Overwhelmed by task	5 (12.8)	6 (22.2)	6 (13.3)	11 (20.4)
Other	8 (20.5)	1 (3.7)	1 (2.2)	12 (22.2)
None	2 (5.1)	0 (0.0)	0 (0.0)	5 (9.3)
15. Which of the following are agency-level barriers that make it harder for you to select and implement evidence-based chronic disease prevention interventions?				
Poor understanding of evidence-based approaches	6 (15.4)	5 (18.5)	4 (8.9)	12 (22.2)
Culture/climate is not supportive of change/new ideas	14 (35.9)	3 (11.1)	1 (2.2)	20 (37.0)
No existing policies to support evidence-based approaches	5 (12.8)	4 (14.8)	14 (31.1)	9 (16.7)
Agency does not provide training in evidence-based approaches	9 (23.1)	10 (37.0)	5 (11.1)	10 (18.5)
Staff/leaders lack formal training in evidence-based approaches	12 (30.8)	7 (25.9)	13 (28.9)	14 (25.9)
Lack of access to resources (e.g., computer, Internet)	4 (10.3)	3 (11.1)	14 (31.1)	3 (5.6)
Not enough funding	22 (56.4)	13 (48.1)	13 (28.9)	40 (74.1)
Low priority placed on chronic disease prevention	5 (12.8)	5 (18.5)	4 (8.9)	12 (22.2)
No systems to ensure interventions are evidence-based	8 (20.5)	16 (59.3)	11 (24.4)	15 (27.8)
Not enough staff	11 (28.2)	4 (14.8)	17 (37.8)	30 (55.6)
Beliefs that evidence-based interventions are too difficult to implement/sustain	4 (10.3)	2 (7.4)	4 (8.9)	4 (7.4)
Other	6 (15.4)	0 (0.0)	0 (0.0)	2 (3.7)
None	3 (7.7)	0 (0.0)	3 (6.7)	3 (5.6)
16. Which of the following are community-level barriers that make it harder for you to select and implement evidence-based chronic disease prevention interventions?				
Lack of access to repositories/databases of scientific studies	7 (17.9)	4 (14.8)	37 (82.2)	7 (13.0)
Lack of partnership between agency and community	13 (33.3)	4 (14.8)	6 (13.3)	13 (24.1)
Community members' needs compete with evidence-based recommendations	22 (56.4)	8 (29.6)	20 (44.4)	30 (55.6)
Catering to preferences of funders^a^	17 (43.6)	3 (11.1)	–	25 (46.3)
Low priority placed on chronic disease prevention	11 (28.2)	14 (51.9)	7 (15.6)	15 (27.8)
Other	4 (10.3)	1 (3.7)	0 (0.0)	6 (11.1)
None	1 (2.6)	7 (25.9)	3 (6.7)	4 (7.4)
17. Which of the following are sociocultural barriers that make it harder for you to select and implement evidence-based chronic disease prevention interventions?				
Distrust of scientific data in the populations served	2 (5.1)	5 (18.5)	7 (15.6)	13 (24.1)
Community cultural practices conflict with evidence-based recommendations	20 (51.3)	9 (33.3)	13 (28.9)	19 (35.2)
Not enough relevant evidence for populations served	18 (46.2)	5 (18.5)	25 (55.6)	14 (25.9)
Serving a rural setting where data are lacking^a^	15 (38.5)	3 (11.1)	–	34 (63.0)
Serving a highly disadvantaged population	18 (46.2)	7 (25.9)	10 (22.2)	21 (38.9)
Serving a population that speaks a language different from the majority^a^	7 (17.9)	1 (3.7)	–	8 (14.8)
Evidence is presented in a language I do not understand	0 (0.0)	1 (3.7)	6 (13.3)	2 (3.7)
Other	1 (2.6)	8 (29.6)	1 (2.2)	2 (3.7)
None	3 (7.7)	2 (7.4)	4 (8.9)	4 (7.4)
18. Which of the following are political/economic barriers that make it harder for you to select and implement evidence-based chronic disease prevention interventions?				
Political leaders not providing enough support	24 (61.5)	10 (37.0)	26 (57.8)	21 (38.9)
Funding changes that occur with changes in political leadership	33 (84.6)	12 (44.4)	13 (28.9)	31 (57.4)
Political climate conflicts with evidence-based chronic disease prevention recommendations	21 (53.8)	2 (7.4)	16 (35.6)	25 (46.3)
Health care system does not support evidence-based chronic disease prevention	15 (38.5)	5 (18.5)	3 (6.7)	15 (27.8)
Other	5 (12.8)	3 (11.1)	0 (0.0)	2 (3.7)
None	1 (2.6)	0 (0.0)	12 (26.7)	5 (9.3)
19. For which of the following skills would you like additional technical support or training:				
Prioritizing program and policy options	6 (15.4)	17 (63.0)	20 (44.4)	16 (29.6)
Quantifying the public health issue using descriptive epidemiology (e.g., concepts of person, place, time)	22 (56.4)	14 (51.9)	15 (33.3)	21 (38.9)
Using quantitative evaluation approaches (e.g., surveillance or surveys)	18 (46.2)	4 (14.8)	14 (31.1)	20 (37.0)
Using qualitative evaluation approaches (e.g., focus groups, key informant interviews)	15 (38.5)	6 (22.2)	11 (24.4)	19 (35.2)
Developing an action plan for achieving goals	13 (33.3)	13 (48.1)	19 (42.2)	17 (31.5)
Defining the health issue according to the community's needs and assets	12 (30.8)	17 (63.0)	26 (57.8)	27 (50.0)
Adapting interventions for different communities and settings	16 (41.0)	16 (59.3)	12 (26.7)	29 (53.7)
Using economic data in the decision making process	21 (53.8)	12 (44.4)	16 (35.6)	22 (40.7)
Communicating research to policy makers	20 (51.3)	8 (29.6)	15 (33.3)	23 (42.6)
Other	1 (2.6)	0 (0.0)	1 (2.2)	1 (1.9)
None	1 (2.6)	7 (25.9)	3 (6.7)	2 (3.7)

a *This item was not applicable and not included in the survey for respondents in China*.

The test-retest reliability coefficients and percentage agreement by question and country appear in Table 3. Of the seven questions with ordinal response options assessed using ICC, six and seven demonstrated good to moderate reliability among practitioners from Australia and the United States, respectively, whereas three questions among practitioners from Brazil and China demonstrated good to moderate reliability. Six of those seven questions were also assessed using percentage agreement. Six and five of the questions demonstrated good to moderate percentage agreement among practitioners from Australia and the United States, respectively, whereas three questions among practitioners from Brazil and one among practitioners from China demonstrated moderate percentage agreement at best.

**Table 3:** Test-retest percent agreement and reliability coefficients by question and country (*N* = 165).

**Questions**	**Australia** **(*****N*** **=** **39)**		**Brazil** **(*****N*** **=** **27)**		**China** **(*****N*** **=** **45)**		**United States** **(*****N*** **=** **54)**	
	**%^**a**^**	**ICC^**b, c**^**	**%**	**ICC**	**%**	**ICC**	**%**	**ICC**
Personal knowledge of evidence-based processes	0.564	0.570	0.667	0.003	0.467	0.511	0.660	0.658
Personal use of repositories to find evidence-based interventions	0.583	0.544	0.630	0.594	0.512	−0.007	0.571	0.508
Workplace staff use of repositories to find evidence-based interventions	0.828	0.762	0.539	0.264	0.576	0.374	0.643	0.515
Percentage workplace programs are evidence-based	–	0.731	–	0.566	–	0.797	–	0.797
Workplace staff use of quality improvement processes	0.581	0.422	0.539	0.423	0.600	0.601	0.600	0.544
Frequency that programs end that should have continued	0.714	0.297	0.444	0.219	0.480	0.174	0.792	0.585
Frequency that programs continue that should have ended	0.828	0.569	0.682	0.116	0.346	−0.094	0.800	0.475
	**Mean %**	**Mean Kappa**^**d**^	**Mean %**	**Mean Kappa**	**Mean %**	**Mean Kappa**	**Mean %**	**Mean Kappa**
Important factors in decision-making related to program planning and implementation, policy development, or funding	0.811	0.305	0.768	0.416	0.739	0.311	0.772	0.361
Avenues used to learn about evidence-based chronic disease prevention interventions	0.774	0.402	0.702	0.297	0.804	0.225	0.786	0.408
Avenues for which additional access is needed	0.810	0.214	0.763	0.154	0.800	0.252	0.778	0.177
Most common reasons for program termination	0.824	0.203	0.693	0.203	0.745	0.179	0.840	0.269
Most common reasons for program continuation	0.758	0.258	0.684	0.219	0.733	0.133	0.754	0.249
Personal barriers to selecting and implementing evidence-based chronic disease prevention interventions	0.823	0.379	0.810	0.308	0.749	0.336	0.857	0.376
Organizational barriers to selecting and implementing evidence-based chronic disease prevention interventions	0.783	0.279	0.790	0.362	0.794	0.260	0.838	0.462
Community-level barriers to selecting and implementing evidence-based chronic disease prevention interventions	0.747	0.235	0.763	0.337	0.836	0.367	0.798	0.387
Sociocultural barriers to selecting and implementing evidence-based chronic disease prevention interventions	0.756	0.286	0.806	0.353	0.808	0.433	0.848	0.476
Political/economic barriers to selecting and implementing evidence-based chronic disease prevention interventions:	0.763	0.204	0.778	0.391	0.747	0.267	0.782	0.329
Skills for which additional technical support or training is needed	0.764	0.466	0.619	0.191	0.687	0.257	0.736	0.273

a *%, Percent agreement*.

b *ICC, Intraclass correlation coefficient*.

a *Survey questions with ordinal response options were assessed using ICC*.

a *Survey questions with a list of response options had each response option dichotomized into selected or not selected, then assessed using Cohen's kappa, and the mean kappa for each set of response options is reported*.

Of the 11 questions whose response options were dichotomized and assessed using mean Cohen's kappa, few questions among practitioners across all four countries showed moderate mean reliability at best (Australia, *N* = 2; Brazil, *N* = 1; China, *N* = 1; United States, *N* = 3). Mean percentage agreement told a different story for these 11 questions. All but one question showed good mean percentage agreement among practitioners from Australia and the United States. Seven and five questions showed good mean percentage agreement among practitioners from Brazil and China, respectively. The remaining of the 11 questions across the countries showed moderate mean percentage agreement.

The following four questions produced less than moderately reliable responses based on both ICC and percentage agreement among practitioners in China: Personal use of repositories to find evidence-based interventions; Workplace staff use of repositories to find evidence-based interventions; Frequency that programs end that should have continued; and Frequency that programs continue that should have ended. Two of those questions (Workplace staff use of repositories to find evidence-based interventions, and Frequency that programs end that should have continued) produced less than moderately reliable responses among practitioners from Brazil based on both measures of reliability as well.

## Discussion

The development and reliability testing of this survey tool are important early steps toward facilitating population-level research that can increase our knowledge of country-specific and cross-country contextual factors that influence the D&I of EBCDP interventions and, in turn, begin to inform more global strategies for improving the D&I of EBCDP. This study, novel in its common methods across countries, showed that the measurement tool produced moderate to good reliability of responses, with at least one measure of reliability, among 14 of the 18 questions across all four countries.

Reliability findings inform the adaptation and further development of this tool. For example, the authors recommend revising the four questions pertaining to personal and workplace staff use of repositories for finding evidence-based interventions and frequency that programs end or continue without warrant before further use among practitioners in China and Brazil. The poor reliability of responses produced from these questions among practitioners from Brazil and China reflect a difference in how they relate to the content of the questions, compared with practitioners from Australia and the United States. This difference may highlight meaningful differences within contexts with respect to D&I processes and structures. For instance, practitioners in countries for which EBCDP is in an earlier stage of dissemination tend to be less knowledgeable about key concepts of EBCDP, making the questions conceptually more difficult and in turn negatively influencing the reliability of their responses (43). Another potential contributing factor to the lower reliability among responses from practitioners in Brazil and China is that the survey tool had to be translated from English to Chinese and Portuguese. Tanzer and Sim review international guidelines on translating and adapting measures across cultural contexts, and this study reflects well the best practices for developing a relevant survey tool for use in the four intended countries (44). For instance, bilingual researchers from each of the four cultural perspectives, as well as public health practitioners working in the chronic disease prevention context in each country were involved in the development of the questions, response options, translations, and reliability testing. Despite steps that the research team took to minimize mis-translation, the meaning of each question and response option becomes one layer removed from its original, intended meaning after translation. Next steps for informing further adaptation of the survey tool should include validity testing among chronic disease prevention practitioners in Australia, Brazil, China, and the United States, ideally in representative samples (45).

There was low prevalence (*N* <5) for many response options and the items with low prevalence varied by country. According to Sim and Wright, low prevalence has stifling effects on Cohen's kappa coefficients, but inflating effects on percentage agreement (46). Low prevalence likely contributed to the low kappa coefficients and comparatively higher percentage agreement found in this study. A larger sample of practitioners across all four countries with more diversity of experiences may improve the variability of responses and the accuracy of reliability findings. Response items with low prevalence of endorsements may also reflect response items that are less applicable to practitioners' experiences in that particular country. Use of this survey tool in a larger, randomly selected sample of chronic disease practitioners in each country would clarify this conjecture.

### Strengths and Limitations

This study responds well to a U.S. federal report that called for additional research focused on the experiences and perspectives of key stakeholders in evidence-based intervention delivery, in order to better facilitate the sustainability of interventions (47). The questions within this survey tool reflect critical contextual factors based on the literature, qualitative interviews of public health practitioners, and expert review (2, 5, 6). This survey tool allows researchers to proceed with research on the D&I of EBCDP interventions on a more global scale than was previously available. To our knowledge, this is the first study of its kind that used common methods across four countries. The research team had particular trouble recruiting retest respondents in Brazil due to significant political unrest that affected public health practitioners at the time of the request (39, 40). This contributed to the longer duration between test and retest and the smaller sample from Brazil compared with the other three countries. Additionally, his survey tool demonstrated lower reliability of responses among practitioners from Brazil and China compared with those from Australia and the United States. Lastly, a convenience sampling approach was carried out in some of the countries to recruit chronic disease prevention practitioners serving local or regional jurisdictions. Such a sampling method introduces potential selection bias and is unlikely to produce representative samples of all chronic disease prevention practitioners in each country. However, the intention of the present study was not to test hypotheses or provide prevalence estimates, which would have required using methods to address sampling error (46). Acknowledging these limitations of the sampling approach, the researcher team ensured that the selected sample included practitioners from various regions of each country, and provided distributions of all survey responses as well as demographic characteristics of the sample.

## Conclusion

This survey tool allows cross-country data collection that can contribute toward an improved understanding of the contextual factors that public health practitioners in Australia, Brazil, China, and the United States face in their daily chronic disease prevention work. This understanding is necessary for the creation of multi-level strategies and policies that promote evidence-based decision-making and effective prevention of chronic diseases on a global scale.

## Ethics Statement

This study was carried out in accordance with the committee responsible for human experimentation (institutional and national) and with the World Medical Association's Declaration of Helsinki with informed consent from all subjects. After reading the electronic informed consent document, subjects indicated their consent by selecting a radial button at the bottom of the informed consent document that read, I consent to participate in this research study. Additional written documentation of consent was waived and the protocol was approved by The University of Melbourne Human Ethics Committee, Pontifica Universidade Catolica do Parana Research Ethics Committee, The Hong Kong Polytechnic University Human Ethics Committee of the Faculty of Health and Social Science, and Washington University in St. Louis Institutional Review Board. Reasons for waived written documentation of consent: Electronic documentation of informed consent was deemed sufficient for this study because of the non-sensitive nature of the questions and the participants' locations in four different countries. The following groups agreed on this decision: The University of Melbourne Human Ethics Committee, Pontifica Universidade Catolica do Parana Research Ethics Committee, The Hong Kong Polytechnic University Human Ethics Committee of the Faculty of Health and Social Science, and Washington University in St. Louis Institutional Review Board.

## Author Contributions

EB contributed to the conception and design of the study, interpretation of data, and drafting of the full manuscript. XY and AdR contributed to the analysis and interpretation of data and drafting of the Statistical Analyses. KS and RB contributed to the conception and design of the study, interpretation of data, and drafting of the Discussion. ZW, PS, TP, RA, and RR contributed to the conception and design of the study. All authors contributed to manuscript revision, read and approved the submitted version.

### Conflict of Interest Statement

The authors declare that the research was conducted in the absence of any commercial or financial relationships that could be construed as a potential conflict of interest.
